# Melorheostosis Involving the Hip Joint

**DOI:** 10.5334/jbsr.3752

**Published:** 2024-10-14

**Authors:** Antoine Vandendriessche, Filip Vanhoenacker

**Affiliations:** 1 Faculty of Medicine, Vrije Universiteit Brussel, Belgium; 2 Department of Radiology, AZ Sint-Maarten, Mechelen, Belgium; 3 Department of Radiology, AZ Sint-Maarten, Mechelen, Belgium; 4 Department of Radiology, UZ Antwerpen, University of Antwerp, Belgium; 5 Department of Radiology, UZ Gent, Faculty of Medicine, Ghent, Belgium

**Keywords:** Melorheostosis, Leri’s disease, Sclerosing bone dysplasia

## Abstract

*Teaching point:* Melorheostosis is a rare sclerosing bone dysplasia, characterized by sclerosis at the periosteal and/or endosteal side of the cortex, causing undulating thickening of the bony contour resembling “dripping candle wax.”

## Case History

**Figure 1:**
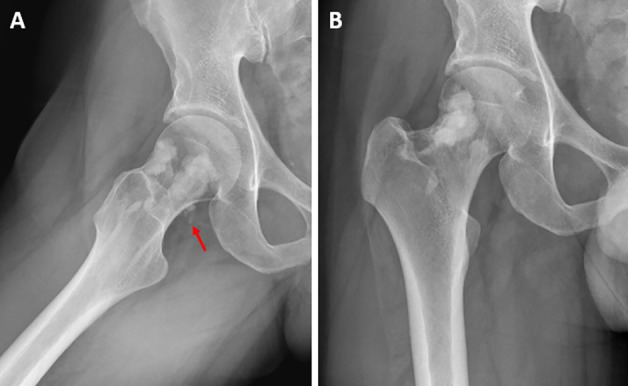
Conventional radiography of the right hip in exorotation **(A)** and AP **(B)**.

**Figure 2:**
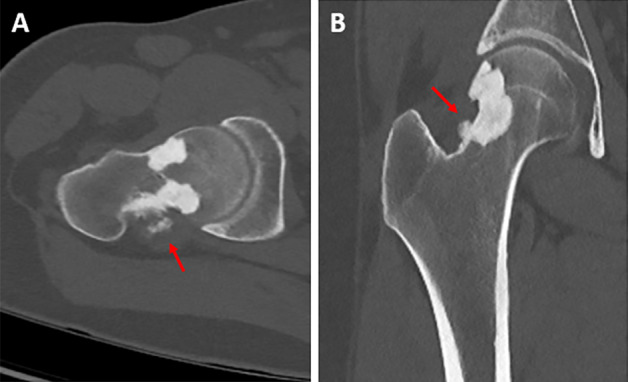
CT of the right hip with axial oblique **(A)** and coronal **(B)** images.

**Figure 3:**
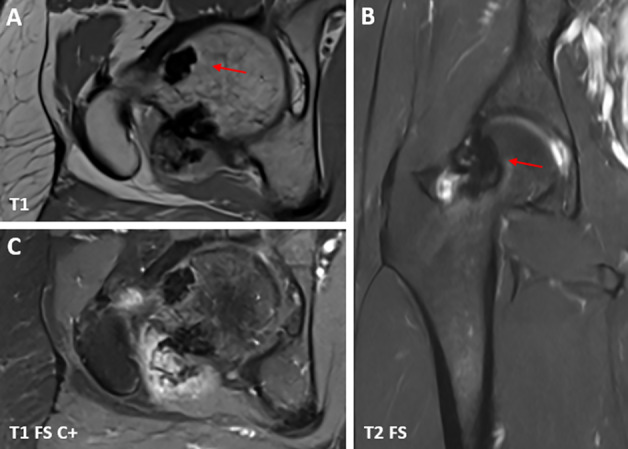
MRI of the right hip with axial **(A)**, coronal **(B)** and axial images after administration of IV‑contrast **(C)**.

A 30‑year‑old man without relevant medical history presented with chronic aspecific pain in the right hip and groin. There was no previous trauma and clinical examination was normal. Conventional radiography (CR) (Figure 1) showed focal sclerosis with multilobular delineation in the neck of the femur with extension into the lateral aspect of the femoral head. The frog leg projection revealed tiny soft tissue calcifications posterior to the femoral neck (Figure 1A, red arrow). Computed tomography (CT) (Figure 2) confirmed endomedullary sclerosis abutting the cortex with subtle excrescences at the lateral femoral head and neck, resembling candle wax dripping. Soft tissue calcifications posterior to the femoral neck were confirmed (Figure 2, red arrows). On magnetic resonance imaging (MRI) (Figure 3), the intramedullary lesions were hypo‑intense on both T1‑ and T2‑weighted images (Figure 3A and 3B, red arrows). There was an absence of enhancement after administration of intravenous gadolinium contrast (Figure 3C). A small articular effusion was seen with mild presumably reactive synovial enhancement.

Based on the typical imaging features, the diagnosis of melorheostosis was made.

## Comments

Melorheostosis, or Leri’s disease, is a sclerosing bone dysplasia characterized by increased bone sclerosis and cortical hyperostosis. The dysplasia is caused by impaired bone breakdown during embryological development [1]. Often, extra‑osseous involvement is seen such as soft tissue calcifications, muscle‑ or tendon contractions, neurovascular anomalies (erythema, oedema or vascular tumours and malformations) and skin disorders [1].

The condition affects men and women equally and can occur at any age, although it is most often detected in teenagers and young adults, as an incidental finding on imaging done for other reasons. The condition usually remains occult until early adulthood and very slow progression is the rule [1]. When symptomatic, pain is generally the first manifestation. Joint stiffness and soft tissue abnormalities may also occur.

The name melorheostosis is derived from the Greek words: melos (limb), rhein (flowing), and ostosis (bone formation). The disease typically affects the appendicular skeleton and can be either monostotic or polyostotic and tends to involve one limb (monomelic), sometimes crossing the joint [1]. The axial skeleton is rarely involved. The sclerosis generally affects one side of the cortex and appears to flow down, similar to candle wax dripping from a candle.

Meticulous analysis of characteristic radiographic features enables a confident diagnosis. CT and MRI are usually not required, although CT may be useful to demonstrate subtle undulation of the cortex and tiny soft tissue calcifications like in our case. Bone and soft tissue lesions are hypo‑intense on all MRI sequences with absence of enhancement.

Treatment for melorheostosis is mainly symptomatic, focusing on pain management and maintaining joint function through physical therapy [1].
